# Clinically adjudicated deceased donor acute kidney injury and graft outcomes

**DOI:** 10.1371/journal.pone.0264329

**Published:** 2022-03-03

**Authors:** Sherry G. Mansour, Nadeen Khoury, Ravi Kodali, Sarthak Virmani, Peter P. Reese, Isaac E. Hall, Yaqi Jia, Yu Yamamoto, Heather R. Thiessen-Philbrook, Wassim Obeid, Mona D. Doshi, Enver Akalin, Jonathan S. Bromberg, Meera N. Harhay, Sumit Mohan, Thangamani Muthukumar, Pooja Singh, Francis L. Weng, Dennis G. Moledina, Jason H. Greenberg, Francis P. Wilson, Chirag R. Parikh

**Affiliations:** 1 Clinical and Translational Research Accelerator, Yale University School of Medicine, New Haven, CT, United States of America; 2 Department of Internal Medicine, Section of Nephrology, Yale University School of Medicine, New Haven, CT, United States of America; 3 Division of Nephrology, Henry Ford Health System, Detroit, MI, United States of America; 4 Department of Medicine, Renal-Electrolyte and Hypertension Division, University of Pennsylvania Perelman School of Medicine, Philadelphia, PA, United States of America; 5 Department of Biostatistics, Epidemiology & Informatics, University of Pennsylvania Perelman School of Medicine, Philadelphia, PA, United States of America; 6 Department of Medical Ethics and Health Policy, University of Pennsylvania Perelman School of Medicine, Philadelphia, PA, United States of America; 7 Division of Nephrology & Hypertension, Department of Internal Medicine, University of Utah School of Medicine, Salt Lake City, UT, United States of America; 8 Division of Nephrology, School of Medicine, Johns Hopkins University, Baltimore, MD, United States of America; 9 Division of Nephrology, Department of Internal Medicine, University of Michigan Medical School, Ann Arbor, MI, United States of America; 10 Montefiore-Einstein Kidney Transplant program, Montefiore Medical Center, Albert Einstein College of Medicine, Bronx, NY, United States of America; 11 Division of Transplantation, Department of Surgery, University of Maryland School of Medicine, Baltimore, MD, United States of America; 12 Department of Microbiology and Immunology, University of Maryland School of Medicine, Baltimore, MD, United States of America; 13 Department of Internal Medicine, Drexel University College of Medicine, Philadelphia, PA, United States of America; 14 Department of Epidemiology and Biostatistics, Drexel University Dornsife School of Public Health, Philadelphia, PA, United States of America; 15 Tower Health Transplant Institute, Tower Health System, West Reading, PA, United States of America; 16 Department of Epidemiology, Columbia University Mailman School of Public Health, New York, NY, United States of America; 17 Division of Nephrology, Department of Medicine, Columbia University Vagelos College of Physicians & Surgeons, New York, NY, United States of America; 18 Division of Nephrology and Hypertension, Department of Medicine, New York Presbyterian Hospital-Weill Cornell Medical Center, New York, NY, United States of America; 19 Department of Transplantation Medicine, New York Presbyterian Hospital-Weill Cornell Medical Center, New York, NY, United States of America; 20 Division of Nephrology, Department of Medicine, Sidney Kimmel Medical College, Thomas Jefferson University Hospital, Philadelphia, PA, United States of America; 21 Saint Barnabas Medical Center, RWJBarnabas Health, Livingston, NJ, United States of America; University of Toledo, UNITED STATES

## Abstract

**Background:**

Acute kidney injury (AKI) in deceased donors is not associated with graft failure (GF). We hypothesize that hemodynamic AKI (hAKI) comprises the majority of donor AKI and may explain this lack of association.

**Methods:**

In this ancillary analysis of the Deceased Donor Study, 428 donors with available charts were selected to identify those with and without AKI. AKI cases were classified as hAKI, intrinsic (iAKI), or mixed (mAKI) based on majority adjudication by three nephrologists. We evaluated the associations between AKI phenotypes and delayed graft function (DGF), 1-year eGFR and GF. We also evaluated differences in urine biomarkers among AKI phenotypes.

**Results:**

Of the 291 (68%) donors with AKI, 106 (36%) were adjudicated as hAKI, 84 (29%) as iAKI and 101 (35%) as mAKI. Of the 856 potential kidneys, 669 were transplanted with 32% developing DGF and 5% experiencing GF. Median 1-year eGFR was 53 (IQR: 41–70) ml/min/1.73m^2^. Compared to non-AKI, donors with iAKI had higher odds DGF [aOR (95%CI); 4.83 (2.29, 10.22)] and had lower 1-year eGFR [adjusted B coefficient (95% CI): -11 (-19, -3) mL/min/1.73 m^2^]. hAKI and mAKI were not associated with DGF or 1-year eGFR. Rates of GF were not different among AKI phenotypes and non-AKI. Urine biomarkers such as NGAL, LFABP, MCP-1, YKL-40, cystatin-C and albumin were higher in iAKI.

**Conclusion:**

iAKI was associated with higher DGF and lower 1-year eGFR but not with GF. Clinically phenotyped donor AKI is biologically different based on biomarkers and may help inform decisions regarding organ utilization.

## Introduction

Less than 20% of patients on the waiting list receive kidney transplants each year, and approximately thirteen patients die every day awaiting a kidney transplant [1]. Despite this unmet demand, 20% of deceased-donor kidneys are discarded, with kidneys from donors with acute kidney injury (AKI) being procured at lower rates and discarded at higher rates [2–4]. Donor AKI usually occurs in the setting of brain-death and significant hemodynamic changes [5]. Brain-death causes loss of spinal cord sympathetic activity leading to vasodilation, impaired cardiac output and hemodynamic instability with reduction in renal perfusion [6,7]. Therefore, increases in serum creatinine concentration in these settings may be due to hemodynamic changes (pre-renal azotemia), rather than intrinsic damage to the kidneys (acute tubular injury). Despite the inability to distinguish between hemodynamic (hAKI) and intrinsic AKI (iAKI) by using serum creatinine alone [8], clinical decisions such as whether to procure or accept a deceased donor kidney are partially determined based on serum creatinine-defined AKI. However, deceased-donor hAKI may be a manifestation of appropriate neurovascular responses to maintain hemodynamic stability [2,9]. Elucidating relationships between types of donor AKI and graft outcomes may help influence allocation decisions. We hypothesize that distinguishing between AKI phenotypes by clinical adjudication will assist in understanding short and long-term graft outcomes.

Multiple studies have shown that deceased donor AKI is not associated with adverse recipient outcomes [10–12]. Lack of these associations may be due to the majority of deceased donors having significant hemodynamic changes leading to functional changes (hAKI) rather than structural injury (iAKI). The importance of phenotyping AKI as hAKI or iAKI is highlighted by literature demonstrating that the two processes are transcriptionally different in the kidney tissue of mouse models, with different tubular injury biomarker concentrations in human urine [13]. In the current study, we determined whether clinical adjudication of deceased donor AKI was associated with recipient outcomes, and whether urine biomarkers distinguish between different phenotypes of AKI.

## Methods

### Study design

This was an ancillary study from the Deceased Donor Study (DDS) and included 428 deceased donors with available charts from two organ procurement organizations (OPOs); Gift of Life Michigan and New York Organ Donor Network. Overall DDS methods have been described in detail elsewhere [14,15]. For the current study, a trained research coordinator manually abstracted seven demographic variables and 50 longitudinal variables from charts of donor hospitalizations from April 2010 to November 2013. Data were managed using a RedCap electronic database. AKI was defined as ≥0.3 mg/dL or ≥50% increase in serum creatinine at any time point during the hospitalization prior to death from the lowest recorded value, irrespective of urine output or duration of time between the two measurements. This corresponded to at least stage 1 AKI by the Acute Kidney Injury Network criteria [16]. We created de-identified donor profiles (**S1A and S1B Fig**) with the following abstracted donor variables: demographics (age, gender, race), daily trends of hemodynamic status (lowest systolic and diastolic pressures, ejection fraction, central venous pressure, PaO2/FiO2 ratio, hemoglobin, vasopressor use) renal function measures (serum creatinine, maximum delta creatinine during hospitalization, blood urea nitrogen-to-creatinine ratio, net fluid balance, urine output, urine casts, urine protein), medications (angiotensin-converting enzyme inhibitors, angiotensin II receptor blockers, vancomycin, diuretics), and microbiology (sputum culture, blood culture, urine culture, bronchial culture). We securely distributed these profiles to three board-certified nephrologists, who independently reviewed AKI cases to adjudicate either as hAKI or iAKI. They were asked to use their clinical judgment to assess the phenotype of AKI based on the donor profiles as they would have done in routine clinical practice. All three nephrologists used common clinical markers such as serum creatinine trends, vital signs, volume status, vasopressor use, and presence of infection to accurately adjudicate the cases. They were blinded to the others’ adjudications, recipient outcomes and study urinary biomarker data. If a nephrologist could not confidently adjudicate hAKI or iAKI, they were asked to label the AKI as mixed subtype (mAKI). Final diagnosis was determined by majority adjudication. If all three nephrologists disagreed, the phenotype was designated as mAKI.

### Biomarker measurement

After collection at time of organ procurement, urine samples were centrifuged at 1000×g for 10 minutes at 4°C, separated into 1 ml aliquots, and immediately stored at -80°C until biomarker measurement. The following urine biomarkers were measured: cystatin-C, albumin-to-creatinine ratio (UACR), interferon alpha (IFN), interleukin (IL-) 4, 6, 8, 10,18, kidney injury molecule-1 (KIM-1), liver-type fatty acid-binding protein (LFABP), neutrophil gelatinase associated lipocalin (NGAL), tumor necrosis factor alpha (TNF-α), chitinase-3-like 1 (YKL-40), epidermal growth factor (EGF), monocyte chemoattractant protein-1 (MCP-1), osteopontin (OPN) and uromodulin (UMOD). NGAL measurement was performed using the Architect platform (Abbott Diagnostics). LFABP was measured using latex-enhanced immunoturbidimetry with anti-human LFABP mouse monoclonal antibodies (Sekisui Medical). All other urine biomarkers were measured using the Meso Scale Discovery platform (MSD, Gaithersburg, MD), which uses electrochemiluminescence detection combined with patterned arrays.

### Operational definitions

Delayed graft function (DGF) in the recipient was defined as the need for any dialysis in the first week post-transplantation. One-year eGFR was calculated by the Chronic Kidney Disease Epidemiology Collaboration equation using the serum creatinine values reported *via* chart review from the DDS cohort [17]. If the recipient died prior to 1 year after transplant, we carried forward their last reported serum creatinine to calculate 1-year eGFR (this occurred in 21 (2%) of recipients who died within the first year of follow up). If the recipient experienced graft failure (GF) prior to 1 year after transplant, 1-year eGFR was imputed as 10 ml/min/1.73m^2^. Finally 1-year GF was defined as return to dialysis or re-transplantation.

### Statistical analysis

All analyses were two-tailed and p-values less than 0.05 were considered significant. Descriptive statistics for continuous variables were reported as median (interquartile range) and for categorical variables as frequencies (%) for the total cohort and stratified by AKI phenotypes. Differences in urine biomarker concentrations and other continuous variables between the three AKI phenotypes were assessed using the Kruskal-Wallis test. Differences in categorical variables including the outcome of GF were assessed using chi-squared test.

When evaluating the association between donor AKI phenotypes and outcomes of DGF and 1-year eGFR, we used non-AKI as the reference group. The associations between AKI phenotypes and the categorical outcome of DGF were analyzed using univariable and multivariable logistic regression clustered at the donor level. The associations between AKI phenotypes and the continuous outcome of 1-year eGFR were analyzed using univariable and multivariable linear regression also clustered at the donor level. Beta (β) coefficients were estimated using the linear regression model, where beta was defined as the change in 1-year eGFR associated with AKI phenotype, when all other variables were held fixed.

Multivariable models were adjusted for the following donor variables that make up the Kidney Donor Profile Index (KDPI): age (years), sex, race, body mass index (BMI), hepatitis C virus (HCV) status, hypertension (HTN), diabetes mellitus (DM), stroke as cause of death, donor donation after cardiovascular determination of death and terminal serum creatinine. In addition to KDPI variables, we adjusted for expanded criteria donor status; transport variables: hypothermic machine perfusion, and cold ischemia time; and recipient variables: age (years), sex, race, DM as the cause of end-stage kidney disease, number of human leukocyte antigen mismatches, panel reactive antibody (%), BMI, pre-emptive transplant status, history of prior kidney transplants and duration of dialysis prior to transplant (months).

In secondary analysis, we evaluated deceased donors having persistent AKI at time of organ procurement defined by an increase in serum creatinine of at least 0.3 mg/dL or 50% increase from the lowest to terminal value. In this subset, we evaluated whether biomarkers measured from urine samples collected at organ procurement differ between AKI phenotypes. Lastly, we also evaluated the associations for AKI phenotypes at time of organ procurement with recipient DGF and 1-year eGFR.

This study used data from the organ procurement and transplantation network (OPTN). The OPTN data system includes data on all donor, wait-listed candidates, and transplant recipients in the US, submitted by the members of OPTN, and has been described elsewhere. The Health Resources and Services Administration, U.S. Department of Health and Human Services provides oversight to the activities of the OPTN contractor. The analyses are based on OPTN data as of January 2017 and may be subject to change due to future data submission or correction by transplant centers. The OPO scientific review committees and the institutional review boards for the participating investigators approved this study under a waiver of consent because deidentified data were used.

## Results

**Fig 1:**
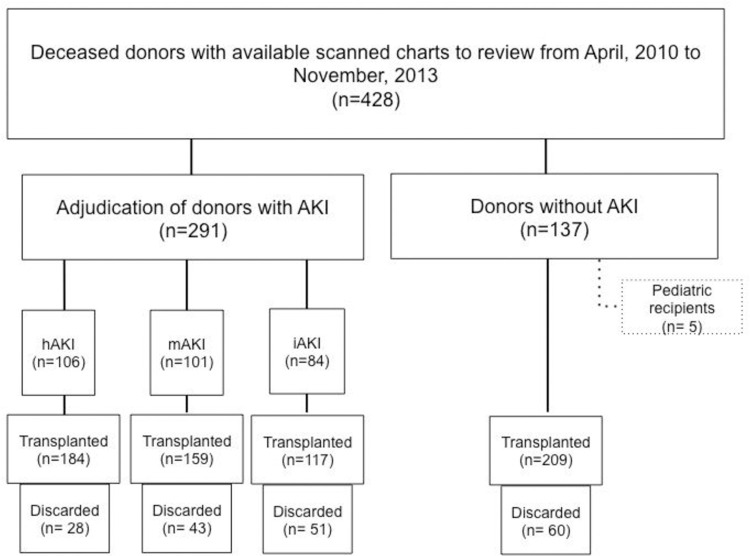
Study flow diagram. Shows the breakdown of our study. A total of 428 donors with available charts were included in our study. Among the 428 donors, 291 had AKI at anytime point during the hospitalization. Only donors with AKI were adjudicated, and 106 were found to have hemodynamic AKI, 101 mixed AKI and 84 intrinsic AKI.

Out of 428 donors, 291 met the clinical AKI definition (**Fig 1**). Among the 291 AKI cases adjudicated, 106 (36%) had hAKI, 101 (35%) had the mAKI, and 84 (29%) had iAKI. Among the adjudicated cases of hAKI, 54 (51%) had perfect agreement (all three nephrologists agreed). Seventeen (17%) cases had perfect agreement in the mAKI subtype 27 (32%) cases had perfect agreement in the iAKI subtype (**S1 Table).** Median donor age was 47 years old (IQR: 31, 57) and 40% were female as shown in **Table 1**. Donor cause of death, KDPI, and admission and terminal serum creatinine significantly differed by AKI phenotype. From the 428 donors evaluated, there were a total of 856 candidate kidneys for donation, with 669 kidney transplanted; 182 kidneys were discarded and 5 kidneys were excluded from the analysis as they were transplanted to pediatric recipients. Rates of discard were,60 (22%), 28 (13%), 43 (21%), and 51 (30%) for no-AKI, hAKI, mAKI and iAKI, respectively (p = 0.004). Recipient characteristics stratified by AKI phenotype are shown in **Table 2.** Recipient age, rate of graft biopsy and hypothermic machine perfusion were significantly higher in the iAKI group, whereas recipient panel reactive antibody was less in iAKI as compared to other groups.

**Table 1:** Donor characteristics by AKI phenotype.

Variables	All (n = 428)	No-AKI (n = 137)	Hemodynamic (n = 106)	Mixed (n = 101)	Intrinsic (n = 84)	P-value
Age (years)	47 (31, 57)	49 (34, 60)	47 (31, 54)	44.5 (28, 54)	45 (33, 56)	0.23
Female	171 (40%)	59 (43%)	46 (43%)	39 (39%)	27 (32%)	0.32
Black Race	80 (19%)	19 (14%)	15(14%)	26 (27%)	20 (24%)	0.05
BMI (kg/m^2^)	28 (24, 32)	27 (24, 32)	28 (24, 32)	27 (23, 33)	29 (24, 32)	0.82
Hypertension	156 (36%)	48 (35%)	37 (35%)	39 (39%)	32 (39%)	0.94
Diabetes	38 (9%)	12 (9%)	14 (15%))	7 (8%)	5 (8%)	0.28
Cause of Death						
Head Trauma	106 (25%)	37 (27%)	28 (27%)	21 (21%)	20 (24%)	0.03
Anoxia	128 (30%)	25 (18%)	36 (34%)	40 (40%)	27 (33%)	
Stroke	177 (41%)	69 (50%)	36 (34%)	38 (38%)	34 (41%)	
Other	11 (3%)	2 (1%)	5 (5%)	2 (2%)	2 (2%)	
Hepatitis C	6 (1%)	0 (0%)	2 (2%)	3 (3%)	1 (1%)	0.28
DCD	60 (14%)	17 (12%)	17 (16%)	11 (11%)	15 (18%)	0.47
KDPI (%)	57 (31, 81)	57 (31, 83)	47 (27, 74)	51 (31, 81)	62 (45, 85)	0.05
Admission sCr (mg/dL)	1.0 (0.8, 1.3)	0.9 (0.7, 1.1)	1.1 (0.8, 1.31)	1.1 (0.9, 1.4)	1.0 (0.8, 1.4)	<0.0001
Terminal sCr (mg/dL)	1.0 (0.7, 1.4)	0.87 (0.7, 1.2)	0.80 (0.6, 1.0)	1.1 (0.7, 1.4)	1.7 (1.2, 3.1)	<0.0001

Values are represented as medians (interquartile ranges) or n(%). Inference testing was done using Kruskal Wallis test for continuous values, and chi-squared test for categorical values. DCD: Donation after cardiovascular determination of death; KDPI: Kidney donor profile index; sCr: Serum creatinine.

**Table 2:** Recipient and transport characteristics by AKI phenotype.

Variables	All (n = 669)	No-AKI (n = 209)	Hemodynamic (n = 184)	Mixed (n = 159)	Intrinsic (n = 117)	P-value
Age (years)	55 (44, 65)	56 (46, 65)	53 (44, 63)	52 (40, 65)	59 (48, 66)	0.05
Female	252 (38%)	84 (40%)	63 (34%)	65 (41%)	40 (34%)	0.43
Black Race	288 (43%)	98 (47%)	71 (39%)	67 (42%)	52 (44%)	0.41
BMI (kg/m^2^)	28 (24, 32)	27 (24, 32)	28 (24, 33)	27 (22, 31)	28 (24, 31)	0.54
Cause of ESKD						0.80
Unknown/other	124 (19%)	32 (15%)	40 (22%)	31 (20%)	21 (18%)	
Diabetes	216 (32%)	76 (36%)	54 (29%)	51 (32%)	35 (30%)	
Hypertension	158 (24%)	50 (24%)	45 (24%)	34 (21%)	29 (25%)	
Glomerulonephritis	111 (17%)	37 (18%)	26 (14%)	29 (18%)	19 (16%)	
Graft Failure	60 (9%)	14 (7%)	19 (10%)	14 (9%)	13 (11%)	
ESKD duration (months)	47 (20,74)	48 (23, 73)	51 (22, 75)	44 (19, 76)	44 (21, 74)	0.73
Pre-emptive transplant	71 (11%)	28 (13%)	21 (11%)	16 (10%)	6 (5%)	0.13
Previous kidney transplant	93 (14%)	23 (11%)	26 (14%)	28 (18%)	16 (14%)	0.35
Recipient PRA						0.01
0%	467 (70%)	151 (72%)	121 (66%)	106 (67%)	89 (76%)	
1–20%	35 (5%)	10 (5%)	12 (7%)	8 (5%)	5 (4%)	
21–80%	84 (13%)	23 (11%)	29 (16%)	18 (11%)	14 (12%)	
>80%	83 (12%)	25 (12%)	22 (12%)	27 (17%)	9 (8%)	
HLA mismatch level	5 (4, 5)	5 (4, 5)	5 (4, 5)	5 (4, 5)	5 (4, 5)	0.31
Kidney biopsied	486 (73%)	168 (72%)	128 (70%)	113 (71%)	98 (84%)	0.03
Hypothermic machine perfusion	503 (75%)	152 (73%)	130 (71%)	122 (77%)	99 (85%)	0.04
Cold Ischemia time (hours)	16 (12, 21)	16 (12, 21)	16 (12, 22)	16 (11, 20)	15 (12, 20)	0.31

Values are represented as medians (interquartile ranges) or n(%). Inference testing was done using Kruskal Wallis test for continuous values, and chi-squared test for categorical values.

BMI: Body mass index; ESKD: End stage kidney disease; HLA: Human leukocyte antigen; PRA: Panel reactive antibody.

Among the 669 transplanted kidneys, 487 (73%) had a procurement biopsy. The rates of biopsies were highest in the iAKI at 98 (84%) vs. 147 (70%), 129 (70%) and 113 (71%) for no-AKI, hAKI and mAKI, respectively (p = 0.03). Among kidneys with biopsies, only 65 (13%) had any acute tubular injury (ATI) reported on biopsy. The presence of any ATI as reported on biopsy (mild or moderate to severe) was not significantly different among the AKI phenotypes [18 (9%) in no AKI, 19 (10%) in hAKI, 14 (9%) in mAKI and 14 (12%) in iAKI, p = 0.91]. The presence of moderate to severe ATI was also not different among the AKI phenotypes [13 (6%) in no AKI, 16 (9%) in hAKI, 14 (9%) in mAKI, and 13 (11%) in iAKI, p = 0.28).

### Distribution of urine biomarkers among AKI phenotypes

A total of 17 biomarkers measured from urine collected at organ procurement were evaluated among non-AKI donors and the three AKI phenotypes happening at anytime during hospitalization. Eight biomarkers were significantly different after indexing to urine creatinine, with NGAL, EGF, cystatin-C, and UMOD having the highest statistically significant differences (**Table 3**). For clinical AKI still ongoing at time of organ procurement, however, ten biomarkers were significantly different- EGF, NGAL, cystatin-C, MCP-1, LFABP, UMOD, UACR, IL-8, YKL-40, and IL-6 (**Table 4**).

**Table 3:** Distribution of biomarkers among AKI phenotypes at anytime during hospitalization.

Urine biomarkers	Total (n = 428)	No AKI (n = 137)	Hemodynamic (n = 106)	Mixed (n = 101)	Intrinsic (n = 84)	P-value
EGF (pg/mg)	5601 (3032, 9541)	5676 (3377, 9385)	8038 (4452, 13132)	6004 (3960, 10218)	2963 (1557, 4710)	<0.001
NGAL (ng/mg)	141 (44.63, 832)	75.34 (26.65, 303)	103 (34.29, 362)	232 (63.97, 1011)	817 (106, 2625)	<0.001
Cystatin C (pg/mg)	2.03 (0.72, 6.93)	1.08 (0.62, 4.56)	1.79 (0.70, 5.24)	2.43 (0.81, 6.52)	4.87(1.11, 13.43)	<0.001
UMOD (ng/mg)	4973 (2426, 13184)	5335 (2505, 10766)	4505 (2326,15008)	7479 (3025,19335)	3703 (1685, 6673)	<0.001
LFABP (ng/mg)	54.83 (13.39, 172.85)	33.80 (8.40, 132.48)	37.83 (15.69, 131.69)	67.80 (10.23, 154.63)	110 (28.57, 296)	0.003
YKL-40 (pg/mg)	2941 (651, 21452)	3167 (871, 18168)	2467 (627, 15367)	1437 (223, 13404)	6335 (986, 128087)	0.008
MCP-1 (pg/mg)	871 (397, 2133	860 (371, 1988)	701 (309, 1925)	795 (394, 1963)	1197 (647, 3742)	0.03
UACR (mg/g)	57.59 (25.85, 139.41)	49.52 (24.21, 126.21)	57.87 (24.77, 132.41)	53.70 (27.95, 105.93)	81.54 (35.42, 252.37)	0.03
IL-8 (pg/mg)	29.81 (9.39, 100.29)	23.04 (7.44, 94.84)	28.63 (6.13, 69.86)	29.06 (7.50, 133.19)	44.09 (15.98, 148.43)	0.06
IL-6 (pg/mg)	3.29 (1.15, 14.25)	3.60 (1.19, 13.02)	2.64 (0.88, 13.63)	2.94 (1.21, 12.25)	7.49 (1.53, 30.36)	0.08
OPN (ng/mg)	2888 (1472, 6719)	3208 (1712, 7215)	2539 (1284, 6480)	2077 (1099, 5729)	3440 (1551, 8405)	0.10
IL-18 (pg/mg)	109.38 (49.97, 304.82)	105.84 (55, 330)	125.78 (56.94, 282.64)	86.14 (45.06, 219)	183 (41.18, 684)	0.16
KIM-1 (pg/mg)	3476 (1804, 6448)	3813 (1974, 7894)	3639 (1972, 6331)	3178 (1409, 6051)	3330 (1599, 5848)	0.32
Creatinine (mg/dL)	43.78 (18.86, 83.41)	45.86 (23.62, 89.89)	41.39 (16.43, 88.56)	37.66 (13.52, 82.22)	44.73 (23.68, 79.80)	0.42
IL-4 (pg/mg)	0.07 (0.04, 0.20)	0.07 (0.04, 0.15)	0.08 (0.03, 0.21)	0.09 (0.04, 0.25)	0.08 (0.04, 0.17)	0.44
TNF-a (pg/mg)	0.56 (0.24, 2.19)	0.44 (0.24, 1.99)	0.66 (0.22, 2.38)	0.63 (0.23, 1.99)	0.60 (0.29, 3.70)	0.56
IL-10 (pg/mg)	0.14 (0.07, 0.34)	0.13 (0.06, 0.29)	0.14 (0.07, 0.27)	0.17 (0.07, 0.48)	0.13 (0.07, 0.27)	0.60
IFN (pg/mg)	2.17 (1.20, 5.86)	2.11 (1.24, 4.29)	2.25 (1.11,5.93)	2.46 (1.18, 7.26)	2.07 (1.23, 4.48)	0.71

Values are represented as medians (interquartile ranges). Inference testing was done using Kruskal Wallis test.

Abbreviations: IFN, interferon alpha; IL, interleukin; KIM-1, kidney injury molecule-1; LFABP, liver fatty acid binding protein; NGAL, neutrophil gelatinase associated lipocalin; TNF, tumor necrosis factor; YKL-40, chitinase 3-like 1; EGF, epidermal growth factor; MCP-1, monocyte chemoattractant protein-1; OPN, osteopontin; UACR: Urine albumin creatinine ratio; UMOD, uromodulin.

**Table 4:** Distribution of biomarkers among AKI phenotypes at time of organ procurement.

Urine biomarkers	Total (n = 428)	No AKI (n = 304)	Hemodynamic (n = 16)	Mixed (n = 45)	Intrinsic (n = 63)	P-value
EGF (pg/mg)	5601 (3032, 9541)	6633 (4334, 11249)	2300 (1342, 8167)	4804 (3433, 6815)	2477 (1308, 3974)	<0.0001
NGAL (ng/mg)	141 (44.63, 832)	90.07 (35.9, 381)	188 (64, 772)	369 (146, 1543)	1103 (239, 2721)	<0.0001
Cystatin C (pg/mg)	2.03 (0.72, 6.93)	1.57 (0.65, 5.31)	1.74 (0.71, 4.31)	3.02 (1.09, 7.79)	7.34 (1.81, 15.61)	<0.0001
MCP-1 (pg/mg)	871 (397, 2133)	708 (349, 1759)	1748 (928, 2647)	962 (443, 2287)	1761 (770, 5246)	<0.0001
LFABP (ng/mg)	54.83 (13.39, 173)	38.46 (9.92, 136.23)	39.46 (22.34, 165.51)	77.13 (28.13, 169.20)	128 (41.78, 356.52)	0.001
UMOD (ng/mg)	4973 (2426, 13184)	5586 (2782, 13692)	2971 (1709, 7099)	6035 (2719, 18258)	3683 (1427, 6673)	0.002
UACR (mg/g)	57.59 (25.85, 139)	54.60 (22.44, 132)	66.94 (30.98, 108)	51.98 (35.79, 93.60)	95.23 (38.85, 267)	0.003
IL-8 (pg/mg)	29.81 (9.39, 100)	24.51 (7.46, 74.69)	39.59 (21.59, 93.49)	23.33 (6.50, 184)	70.39 (25.30, 189)	0.005
YKL-40 (pg/mg)	2941 (651, 21452)	2433 (682, 14555)	3249 (370, 69635)	2188 (156, 17820)	16037 (1650, 204203)	0.006
IL-6 (pg/mg)	3.29 (1.15, 14.25)	2.80 (1.07, 11.96)	4.49 (2.05, 10.38)	3.73 (1.05, 12.71)	11.13 (1.79, 39.16)	0.006
IL-18 (pg/mg)	109 (49.97, 305)	106 (48.96, 231)	245 (61.19, 594)	89.54 (52.56, 220)	219 (47.04, 771)	0.12
KIM-1 (pg/mg)	3476 (1804, 6448)	3466 (1826, 6489)	5327 (3479, 8527)	2795 (1093, 6366)	3528 (1599, 5848)	0.18
TNF-a (pg/mg)	0.56 (0.24, 2.19)	0.52 (0.23, 2.12)	0.45 (0.21, 0.78)	0.72 (0.23, 2.71)	0.63 (0.33, 3.89)	0.19
IL-4 (pg/mg)	0.07 (0.04, 0.20)	0.04 (0.04, 0.20)	0.07 (0.03, 0.10)	0.09 (0.03, 0.25)	0.09 (0.05, 0.24)	0.34
Creatinine (mg/dL)	43.78 (18.86, 83.41)	44.40 (18.51, 86.22)	53.17 (40.88, 112)	42.59 (13.20, 89.38)	39.02 (20.06, 71.36)	0.51
IFN (pg/mg)	2.17 (1.20, 5.86)	2.11 (1.18, 5.65)	2.10 (0.77, 3.29)	2.37 (1.05, 7.64)	2.47 (1.50, 6.09)	0.56
OPN (ng/mg)	2888 (1472, 6719)	2828 (1476, 6530)	2886 (1620, 6032)	2062 (1099, 8986)	3436 (1526, 8405)	0.73
IL-10 (pg/mg)	0.14 (0.07, 0.34)	0.14 (0.07, 0.35)	0.13 (0.06, 0.20)	0.15 (0.06, 0.60)	0.17 (0.08, 0.28)	0.78

Values are represented as medians (interquartile ranges). Inference testing was done using Kruskal Wallis test.

Abbreviations: ACR: Albumin creatinine ratio; IFN, interferon alpha; IL, interleukin; KIM-1, kidney injury molecule-1; LFABP, liver fatty acid binding protein; NGAL, neutrophil gelatinase associated lipocalin; TNF, tumor necrosis factor; YKL-40, chitinase 3-like 1; EGF, epidermal growth factor; MCP-1, monocyte chemoattractant protein-1; OPN, osteopontin; UACR: Urine albumin creatinine ratio; UMOD, Uromodulin.

### Associations of AKI phenotypes at anytime during hospitalization with DGF

**Fig 2:**
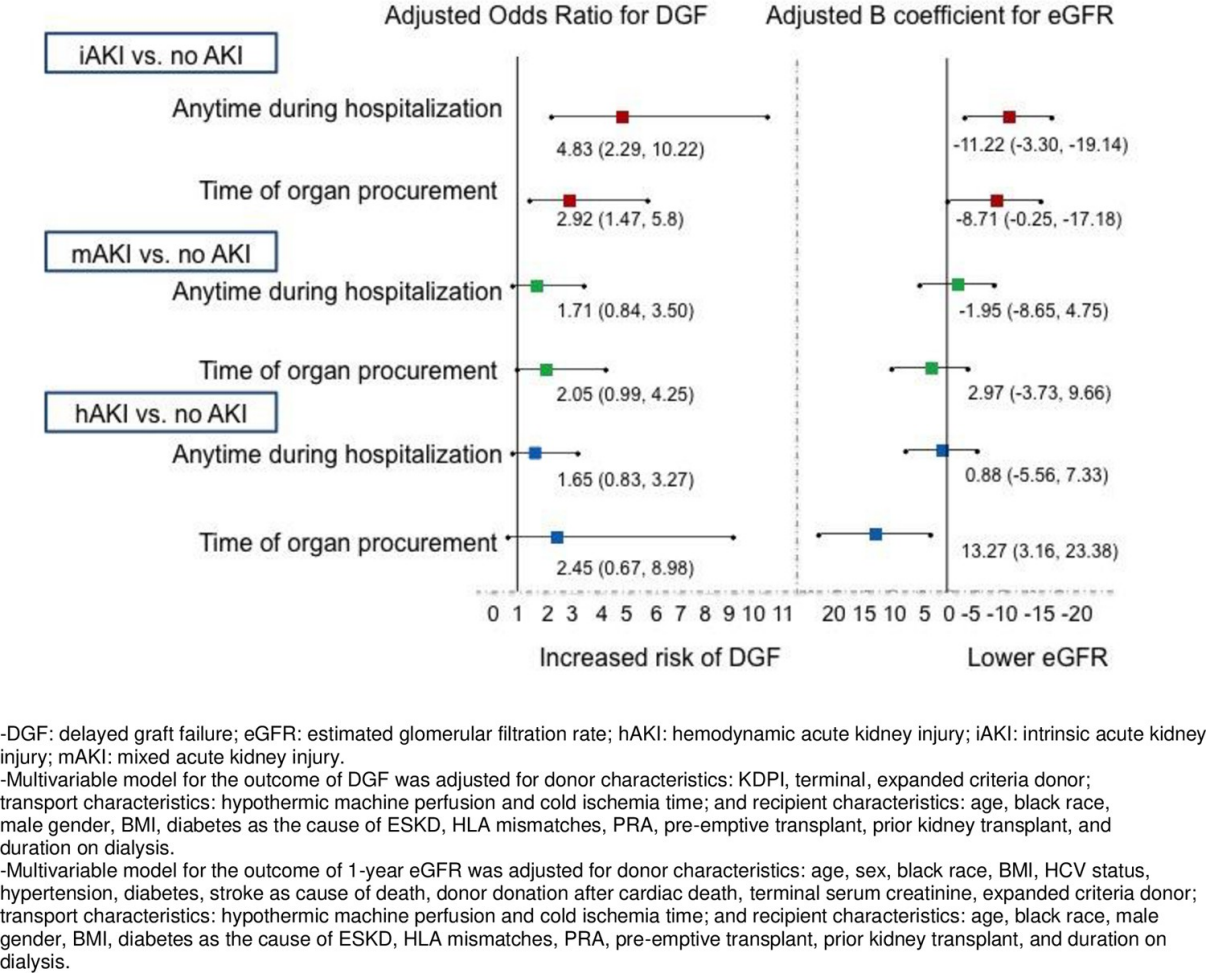
Associations of AKI phenotypes with DGF and 1-year eGFR. Shows the independent associations between AKI phenotypes as compared to hemodynamic AKI and the outcomes of delayed graft function and 1-year eGFR. The exposure of AKI phenotypes is shown both as defined by AKI happening anytime during donor hospitalization as well as AKI at time of organ procurement.

Out of 669 kidneys transplanted, 209 were from non-AKI donors, 184 were from donors with hAKI, 159 from donors with the mAKI and 117 from donors with iAKI. DGF occurred in 216 (32%) kidneys, with the highest rate of DGF in kidneys from donors with iAKI, 60 (51%), followed by hAKI, 59 (32%), mAKI, 44 (28%), and non-AKI, 53 (25%), p<0.0001. In univariable analyses, iAKI had significantly increased odds of DGF compared to non-AKI, but neither hAKI nor mAKI were significantly associated with DGF as shown in **Table 5 and Fig 2**. Adjusting for donor and recipient characteristics, the associations remained significant with iAKI having 5 times the odds of DGF compared to non-AKI [aOR (95% CI): 4.83 (2.29, 10.22)]. There were no significant associations with DGF when comparing the hAKI and mAKI with non-AKI. Full multivariable model is shown in **S2 Table**.

**Table 5:** Associations between AKI phenotypes and DGF and 1-year eGFR.

**Variables**	**Event Rate in Recipients (n/total)**	**Univariable [OR (95% CI)]**	**Multivariable** ^**a**^ **[OR (95% CI)]**
Association between AKI Phenotypes at anytime during hospitalization and DGF			
No AKI	53/209 (25%)	(ref)	(ref)
hAKI	59/184 (32%)	1.39 (0.87, 2.23)	1.65 (0.83, 3.27)
mAKI	44/159 (28%)	1.13 (0.67, 1.88)	1.71 (0.84, 3.50)
iAKI	60/117 (51%)	3.10 (1.87, 5.13)	4.83 (2.29, 10.22)
	**Median (IQR) of 1-year eGFR**	**Univariable [B coefficient (95% CI)]**	**Multivariable** ^**b**^ **[B coefficient (95% CI)]**
Association between AKI Phenotypes at anytime during hospitalization and 1-year eGFR			
No AKI		(ref)	(ref)
hAKI		0.93 (-4.60, 6.47)	0.88 (-5.56, 7.33)
mAKI		1.01 (-5.04, 7.06)	-1.95 (-8.65, 4.74)
iAKI		-4.82 (-10.62, 0.97)	-11.22 (-19.14, -3.30)

AKI: Acute kidney injury; DGF: Delayed graft function; eGFR: Estimated glomerular filtration rate.

^a^ Multivariable model was adjusted for donor characteristics: KDPI, expanded criteria donor; transport characteristics: Hypothermic machine perfusion and cold ischemia time; and recipient characteristics: Age, black race, male gender, BMI, diabetes as the cause of ESKD, HLA mismatches, PRA, pre-emptive transplant, prior kidney transplant, and duration on dialysis.

^b^ Multivariable model was adjusted for donor characteristics: Age, sex, black race, BMI, HCV status, hypertension, diabetes, stroke as cause of death, donor donation after cardiac death, terminal serum creatinine, expanded criteria donor; transport characteristics: Hypothermic machine perfusion and cold ischemia time; and recipient characteristics: Age, black race, male gender, BMI, diabetes as the cause of ESKD, HLA mismatches, PRA, pre-emptive transplant, prior kidney transplant, and duration on dialysis.

### Associations of AKI phenotypes at anytime during hospitalization with 1-year eGFR

One-year eGFR was numerically lower in iAKI {iAKI [median (IQR) of 49 (35, 67) mL/min/1.73m^2^], compared to non-AKI [52 (43, 69) mL/min/1.73m^2^], mAKI [55 (43, 74) mL/min/1.73m^2^] and hAKI [57 (39, 72) mL/min/m^2^], p = 0.22} but did not reach statistical significance. On multivariable analysis, iAKI was independently associated with an 11 ml/min/1.73m^2^ decrease in eGFR compared to non-AKI [adjusted B coefficient (95% CI): -11.22 (-19.14, -3.30)] as shown in **Table 5 and Fig 2**. The full multivariable model is shown in **S3 Table**. DGF was not a significant effect modifier for the association between AKI phenotypes and 1-year eGFR.

### Rates of 1-year GF among AKI phenotypes

A total of 34 recipients experienced GF by 1-year. The distribution of GF among AKI phenotypes was not significantly different. Among recipients of kidneys from non-AKI donors, 3.8% (8/209) developed GF. Among recipients of kidneys from donors with AKI, 4.3% (8/184) developed GF in the hAKI group, 6.3% (10/159) in the mAKI group, and 6.8% (8/117) in the iAKI group. These rates were not significantly different, p = 0.32.

### Associations of AKI phenotypes at time of procurement with DGF and 1-year eGFR

We evaluated a subset of donors with persistent AKI at time of organ procurement, and out of 291 donors with AKI during hospitalization, 124 (43%) had persistent AKI at organ procurement. Of these, 16 (13%) had hAKI, 45 (36%) had mAKI and 63 (51%) had iAKI. Compared to non-AKI, iAKI was associated with nearly 3-fold odds of having DGF [2.92 (1.47, 5.8)] and lower 1-year eGFR [-8.71 (-0.25, -17.18) ml/min/1.73m^2^ (**Fig 2**). In contrast hAKI was associated with higher 1-year eGFR [13.27 (3.16, 23.38) ml/min/1.73m^2^] compared to non-AKI, but was not associated with DGF. mAKI was not associated with DGF or 1-year eGFR.

## Discussion

We evaluated associations between clinically adjudicated deceased-donor AKI and recipient outcomes in this multicenter study. We found that clinically phenotyped deceased-donor AKI had biological differences as evidenced by urine injury and repair biomarkers. We also found that donor iAKI happening earlier during donor hospitalization or ongoing at organ procurement was significantly associated with increased risk of DGF and lower 1-year eGFR but was not associated with early GF.

Our study further explores the biological differences between hAKI and iAKI as identified by Barasch et al [13], and contributes to the argument that the sole reliance on serum creatinine, without phenotyping AKI, neglects relevant prognostic data that associate with graft outcomes [18,19]. To our knowledge, this study is first to demonstrate that this biological difference, as measured by urine biomarkers, exists within clinically adjudicated deceased-donor AKI phenotypes. Current diagnostic strategies such as fractional excretion of sodium (FeNa) and urea are often unable to make the distinction between structural (iAKI) and functional (hAKI) disease. Many studies have reported FeNa <1% in iAKI, and although it has moderate discrimination for iAKI, its sensitivity and specificity decrease in patients using diuretics [20–22]. Consequently clinicians are left to rely on retrospective data such as response to fluids to differentiate between hemodynamic and intrinsic etiologies of AKI [23,24]. Our findings validate physicians’ clinical acumen and highlight certain urine biomarkers as targets for future research to distinguish between AKI phenotypes and to limit subjectivity from this clinically challenging setting. Furthermore, our findings that iAKI is more highly associated with DGF and lower eGFR suggest that phenotyping AKI is important for predicting recipient outcomes. More so, these findings may offer an opportunity for treating clinicians to modify certain risk factors leading to iAKI in donors prior to organ procurement such as avoidance of hypotension, and treatment of any infections. This also highlights the importance of assessing the etiology and phenotype of AKI prior to organ acceptance or rejection.

Additionally, clinically adjudicated iAKI was associated with increased risk for DGF and lower 1-year eGFR, further highlighting the potential benefit of accurately phenotyping AKI. hAKI is a functional change in the kidneys with reduction in filtration, but iAKI involves tubular cell injury and structural damage to the kidneys [25–27]. Given the pathophysiologic, and known transcriptional differences in kidney tissue between intrinsic and hemodynamic AKI [13], it is biologically plausible that iAKI in deceased donors is associated with an increase in DGF and lower 1-year eGFR. Furthermore, our findings are in agreement with prior literature, which shows that tubular injury on histology is associated with DGF [28,29]. Our results also highlight that phenotyping AKI both clinically and by biomarkers is important in terms of recipient outcomes. Pre-procurement identification of donors with iAKI using biomarkers such as NGAL may offer a window for clinicians to intervene to improve future recipient outcomes. We previously identified that urine NGAL among other biomarkers was not associated with recipient outcomes [15]. However, urine NGAL has been shown to be associated with ATI severity in deceased donors [30]. Our current findings suggest that NGAL may potentially have a different association with recipient outcomes in the setting of iAKI. Future studies with larger sample size will need to investigate this further.

When evaluating GF, our study was limited by sample size but did not identify differing rates of GF among clinically adjudicated AKI phenotypes. Although future studies with larger sample size are needed to properly investigate the potential association between iAKI and GF, the findings of our study suggest that donor iAKI may lead to significantly lower 1-year eGFR but this decline in graft function may not be clinically meaningful to manifest as graft failure. These findings are consistent with our previously published data, which have shown that deceased-donor AKI, defined by terminal serum creatinine, is not associated with GF [9]. This lack of association with GF is likely due to the unique events surrounding deceased-donor AKI and could be partially explained by the predominance of hAKI among deceased donors as we have shown in this study. Labeling deceased-donor AKI as one disorder by a rise in serum creatinine rather than a heterogeneous condition and manifestation of multiple disorders, risks the potential discard of kidneys with good transplant prognosis. In fact, our study identified a subset of donors with ongoing hAKI at time of organ procurement with better 1-year graft function as compared to non-AKI.

Our findings need to be interpreted in the context of our study’s limitations. The three adjudicators may not be an accurate representation of the general physician population as all trained at the same institution. The phenotyping of AKI was mainly as nephrologists were encouraged to use their clinical judgment. However, this more accurately reflected real life clinical settings, as physicians rely on their clinical acumen to classify and phenotype AKI. Another limitation in our study involved our definition of AKI, which was based on a rise in serum creatinine, and did not account for potential creatinine level fluctuations in undiagnosed chronic kidney disease in the donors. In addition, the biomarker differences among phenotypes could have captured the clinical severity rather than the actual etiology of AKI as adjudicators assessed a wide variety of variables including laboratory, medication, as well as demographic data to adjudicate donor AKI cases. Histological confirmation of our clinically adjudicated AKI phenotypes was limited as ATI on biopsy was only found in <15% of kidneys in our study. This is limited by some practical concerns as procurement wedge biopsies are usually interpreted in a rush by non-renal pathologists, and hence tubular injury may not be accurately reported [31]. However, the absence of a relationship between the evidence of ATI on biopsy and clinical AKI phenotypes further calls into question the utility of procurement biopsies [32–34]. Alternatively, biomarkers such as NGAL have been shown to be specific to tubular injury in the kidneys, which we have shown to be significantly higher in the iAKI group [35]. Furthermore, our AKI definition utilized lowest serum creatinine as the baseline and a change of 0.3 mg/dL could have preceded the lowest creatinine measurement. This approach presumes that some donors could have incurred AKI prior to admission and that admission creatinine is not representative of their baseline value. Given the inclusivity of this definition, less severe AKI could have been included in our cohort. Another limitation is the lack of adjustment for multiple comparisons for the number of biomarkers and clinical variables tested. Lastly, our results need to be validated in a larger sample size. Future studies may take an alternative approach with a focus on machine learning techniques and data-driven approaches to identify variables predictive of clinically adjudicated AKI in a smaller subset, which can then be applied to larger subsets to assess the validity of our findings [36].

In conclusion, we have shown that clinically adjudicated deceased-donor hAKI and iAKI were biologically different by injury and repair urine biomarkers. iAKI was associated with higher rates of DGF and lower 1-year eGFR but was not associated with GF, whereas higher 1-year eGFR was noted for kidneys with hAKI at time of organ procurement. Clinically phenotyped deceased donor AKI may help inform decisions regarding organ allocation and utilization.

## Supporting information

S1 Fig **a:** Example of a Deceased-Donor Profile (adjudicated as hemodynamic AKI by all three adjudicators). We created de-identified donor profiles abstracted donor clinical variables and distributed these profiles to nephrologists for adjudication. **b:** Example of a Deceased-Donor Profile (adjudicated as intrinsic AKI by all three adjudicators). We created de-identified donor profiles abstracted donor clinical variables and distributed these profiles to nephrologists for adjudication.
(TIF)

S1 Table Breakdown of agreement among nephrologists. Among the adjudicated cases of hAKI, 51% had perfect agreement in the hAKI subtype, 17% had perfect agreement in the mAKI subtype and 32% had perfect agreement in the iAKI subtype.
(TIF)

S2 Table Full multivariable model for the outcome of DGF. There were no significant associations with DGF when comparing the hAKI and mAKI with non-AKI.
(TIF)

S3 Table Full multivariable model for the outcome of 1-year eGFR. iAKI was independently associated with an 11 ml/min/1.73m^2^ decrease in eGFR compared to non-AKI.
(TIF)
