# Supplementary figures and images for: Clinically adjudicated deceased donor acute kidney injury and graft outcomes
Source: PLoS One. 2022 Mar 3;17(3):e0264329. doi: 10.1371/journal.pone.0264329 (PMC8893682; doi:10.1371/journal.pone.0264329)

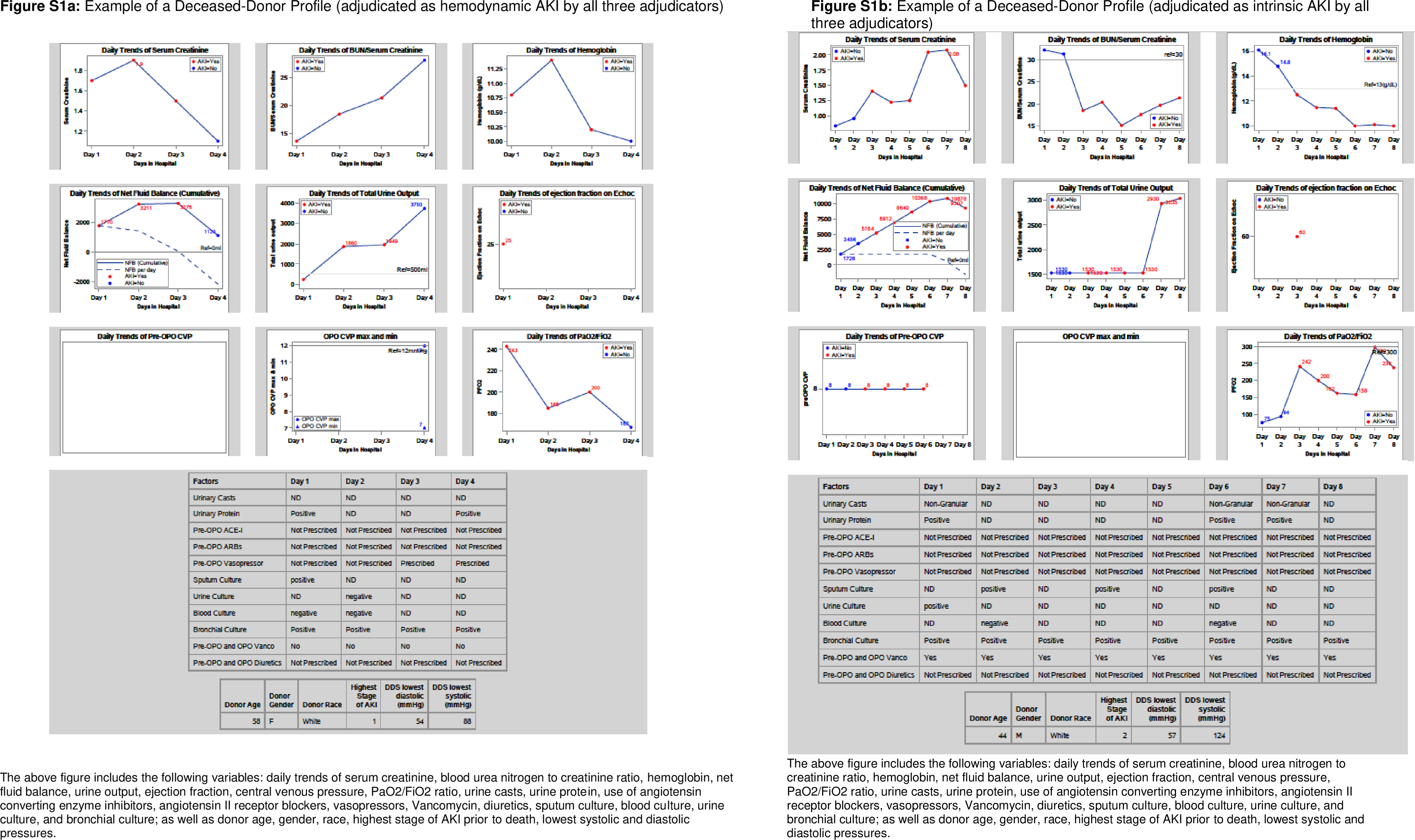

Supplement: S1 Fig — a: Example of a Deceased-Donor Profile (adjudicated as hemodynamic AKI by all three adjudicators). We created de-identified donor profiles abstracted donor clinical variables and distributed these profiles to nephrologists for adjudication. b: Example of a Deceased-Donor Profile (adjudicated as intrinsic AKI by all three adjudicators). We created de-identified donor profiles abstracted donor clinical variables and distributed these profiles to nephrologists for adjudication. (TIF) [file pone.0264329.s001.tif]

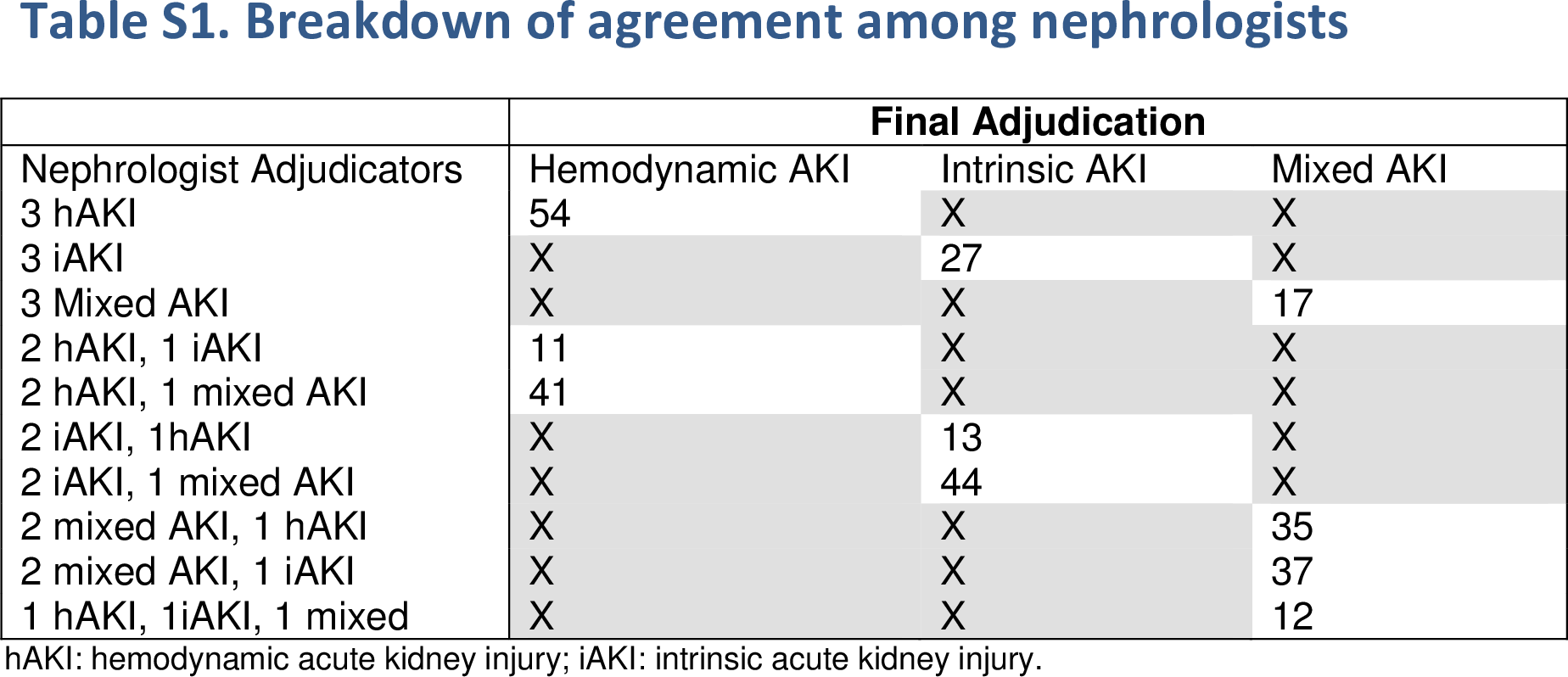

Supplement: S1 Table — Among the adjudicated cases of hAKI, 51% had perfect agreement in the hAKI subtype, 17% had perfect agreement in the mAKI subtype and 32% had perfect agreement in the iAKI subtype. (TIF) [file pone.0264329.s002.tif]

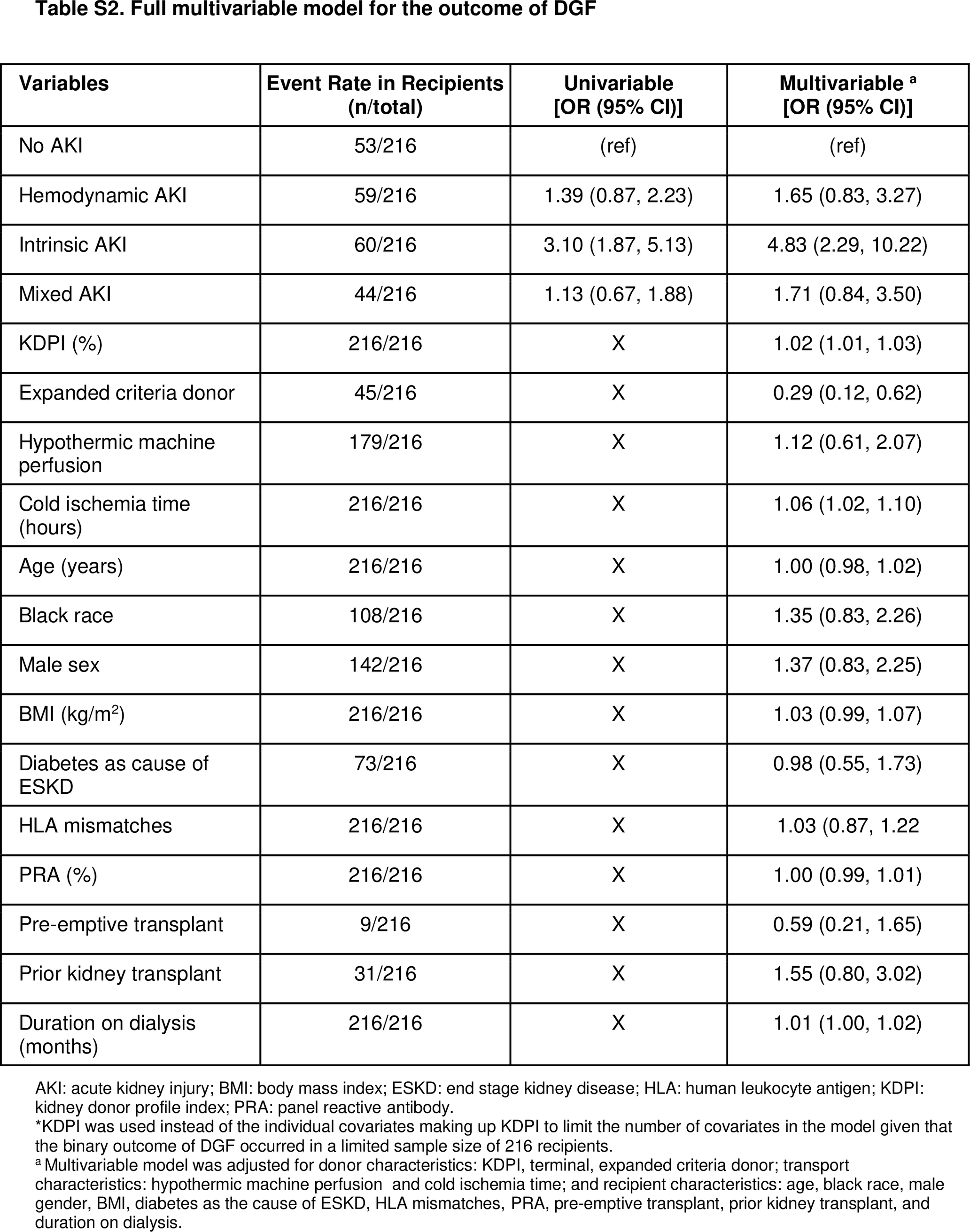

Supplement: S2 Table — There were no significant associations with DGF when comparing the hAKI and mAKI with non-AKI. (TIF) [file pone.0264329.s003.tif]

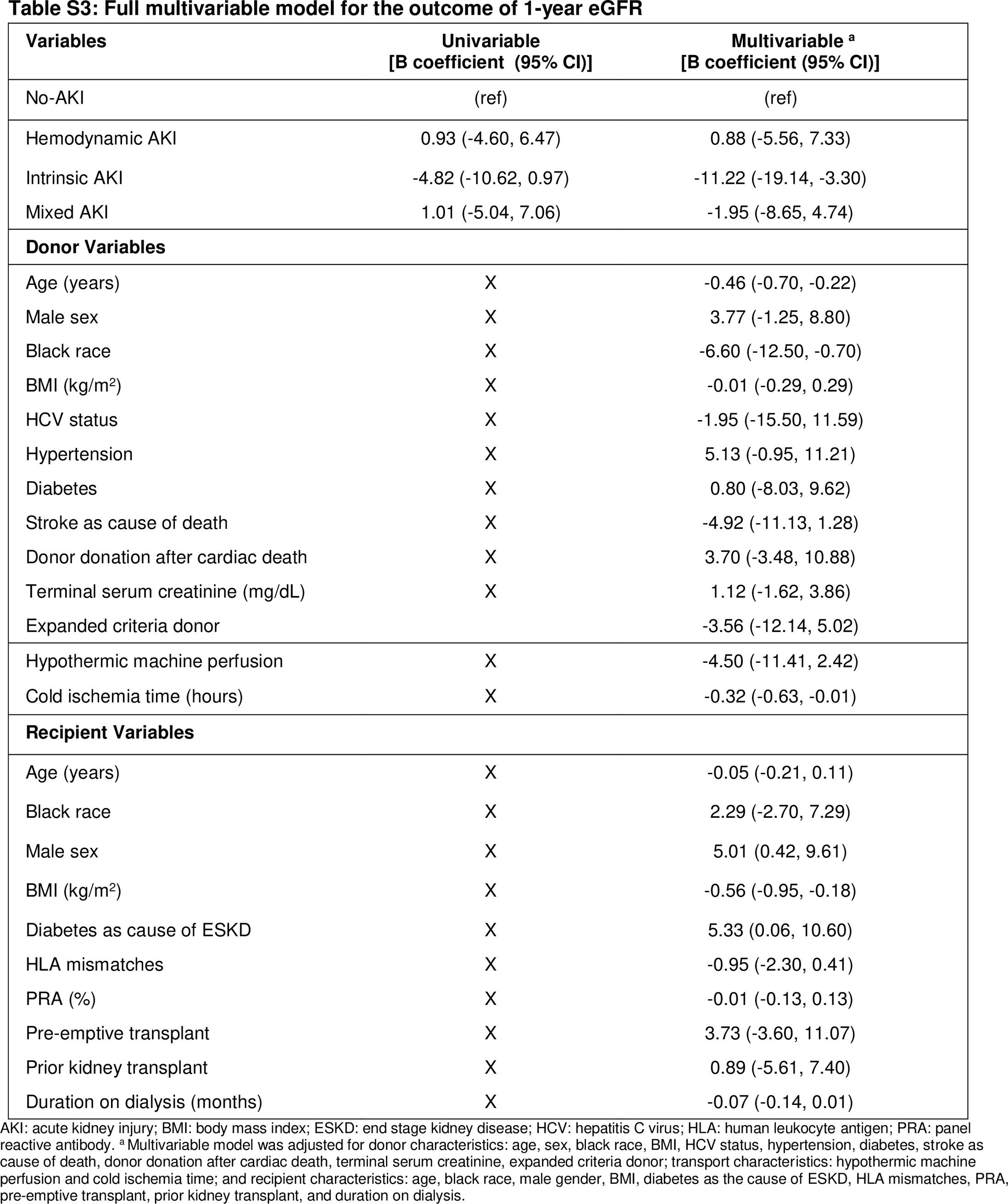

Supplement: S3 Table — iAKI was independently associated with an 11 ml/min/1.73m2 decrease in eGFR compared to non-AKI. (TIF) [file pone.0264329.s004.tif]
